# Uterine choriocarcinoma coexistence with endometroid adenocacinoma: a case report and literature review

**DOI:** 10.1186/s12905-023-02377-1

**Published:** 2023-05-10

**Authors:** Liping Bai, Yali Chen, Ling Han, Ai Zheng, Xiaoyu Mo

**Affiliations:** 1 Department of Gynaecology and Obstetrics, West China Second Hospital, Sichuan University, 3 Section of People South Street, Chengdu, 610041 P. R. China; 2 Key Laboratory of Birth Defects and Related Diseases of Women and Children, Ministry of Education, Sichuan University, Chengdu, Sichuan 610041 P. R. China

**Keywords:** Choriocarcinoma, Endometrial carcinoma, Coexist, Case report

## Abstract

**Background:**

Choriocarcinoma coexisting with endometrial carcinoma is rare. To the best of our knowledge, only one case of choriocarcinoma coexisting with endometrial carcinoma has been reported.

**Case presentation:**

Here, we present this case and provide a literature review. A 38-year-old unmarried nulliparous woman presented to the clinic with a menstrual disorder for more than 3 months. She then underwent a hysteroscopic procedure. The pathological findings were malignant, two types of carcinoma, and no transitional lesions were observed; about 85% of them were choriocarcinoma with smooth muscle infiltration and intravascular investigation of the thrombus; about 15% were highly differentiated endometrioid adenocarcinoma; Immunohistochemistry (endometrioid/choriocarcinoma): Vim (+ + / +  + +), P40 (+ ±), CK5/6 multifocal ( ±), CK7 ( ±), EMA (+ ±), P16 multifocal ( ±), P53 (+ / + +), WT-1 (-/ + +), hCG (-/ +  + +), CD138 (-/ +  + +), Gly-3 (-/-), ER ( ±), PR (+ ±), Sall-4 (-/-), P21 (-/ +), P27 (-/ +  + +), CyclinE (-/ + +), Ki67 positivity rate (10%/95%). We performed a laparoscopic hysterectomy, bilateral adnexectomy, and pelvic and para-abdominal lymph node dissection after five cycles of chemotherapy. She was diagnosed with choriocarcinoma with endometrial cancer, stage IVb choriocarcinoma and stage IA endometrial cancer. Postoperative radiochemotherapy was administered. The patient was disease-free 40 months after the treatment ended.

**Conclusion:**

We report a case of choriocarcinoma coexisting with endometrial carcinoma and provide a literature review that may help inspire additional studies in the future.

## Introduction

Choriocarcinomas are composed of mononucleated and multinucleated trophoblasts and are categorized as gestational or non-gestational carcinoma. Gestational choriocarcinoma occurs after a previous gestational event while non-gestational choriocarcinoma occurs in patients with no previous pregnancy history and typically exists as a mixed component of germ cell tumors and carcinomas. Endometrial carcinoma(EC) is the third most common gynecological cancer, tertiary to cervical and ovarian cancers. Approximately 10% of ECs have mixed histology, comprising ≥ 10% of other components, and often occurs in individuals aged > 75 years [1]. To the best of our knowledge, only one case of choriocarcinoma coexisting with EC has been reported [2], implying its rarity. Here, we present this case and provide a literature review.

## Case presentation

**Fig. 1:**
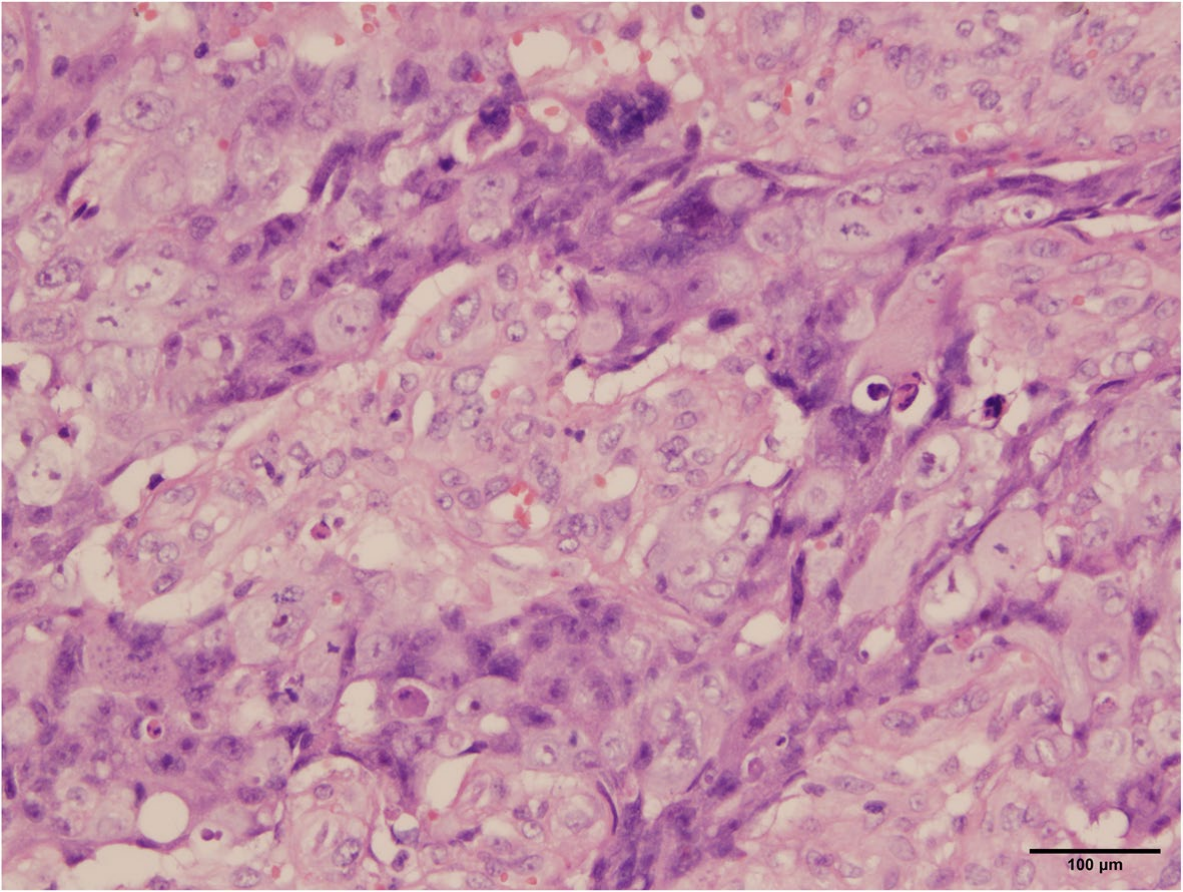
Histopathological resultsidentified the mass as choriocarcinoma (200 ×)

**Fig. 2:**
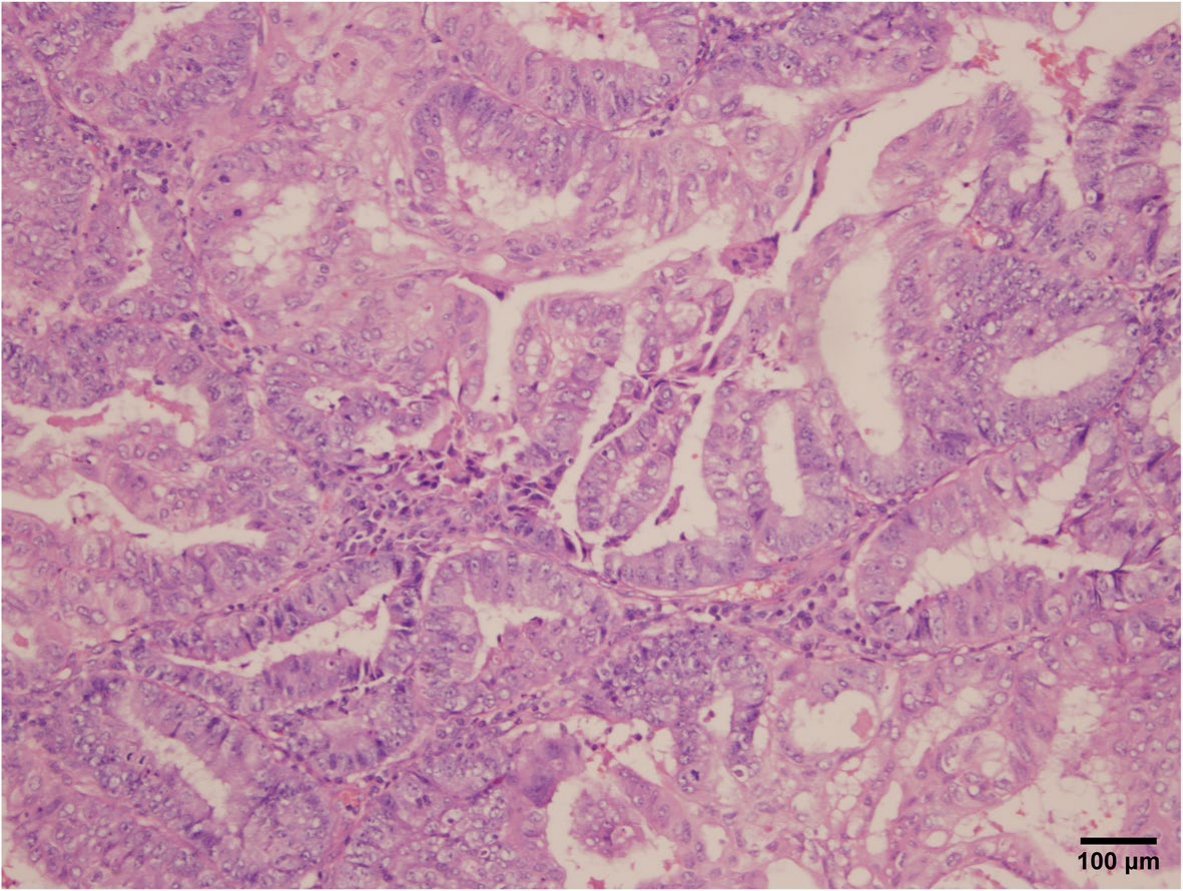
The curettage tissue pathological results indicated endometrioid carcinoma (100 ×)

**Fig. 3:**
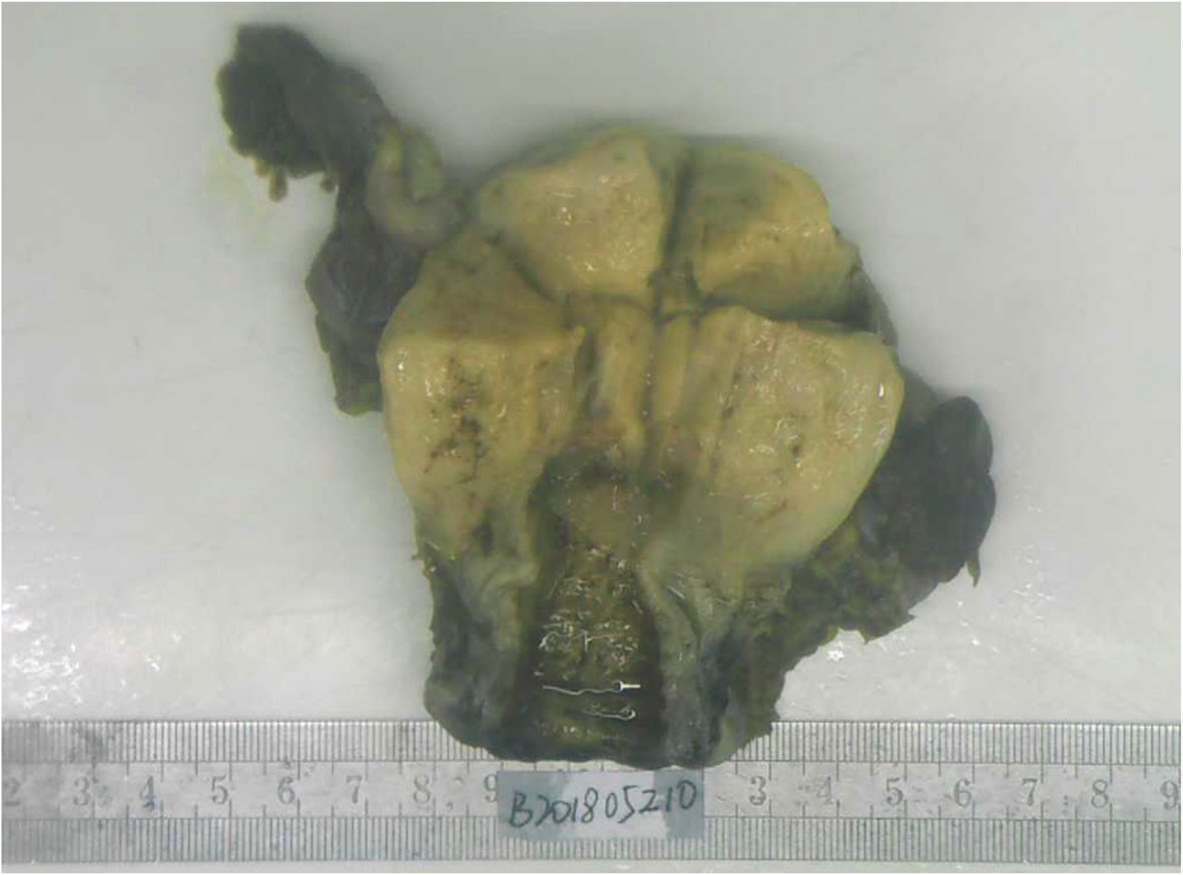
Gross surgical specimen of the uterus showed no obvious lesions after chemotherapy

**Fig. 4:**
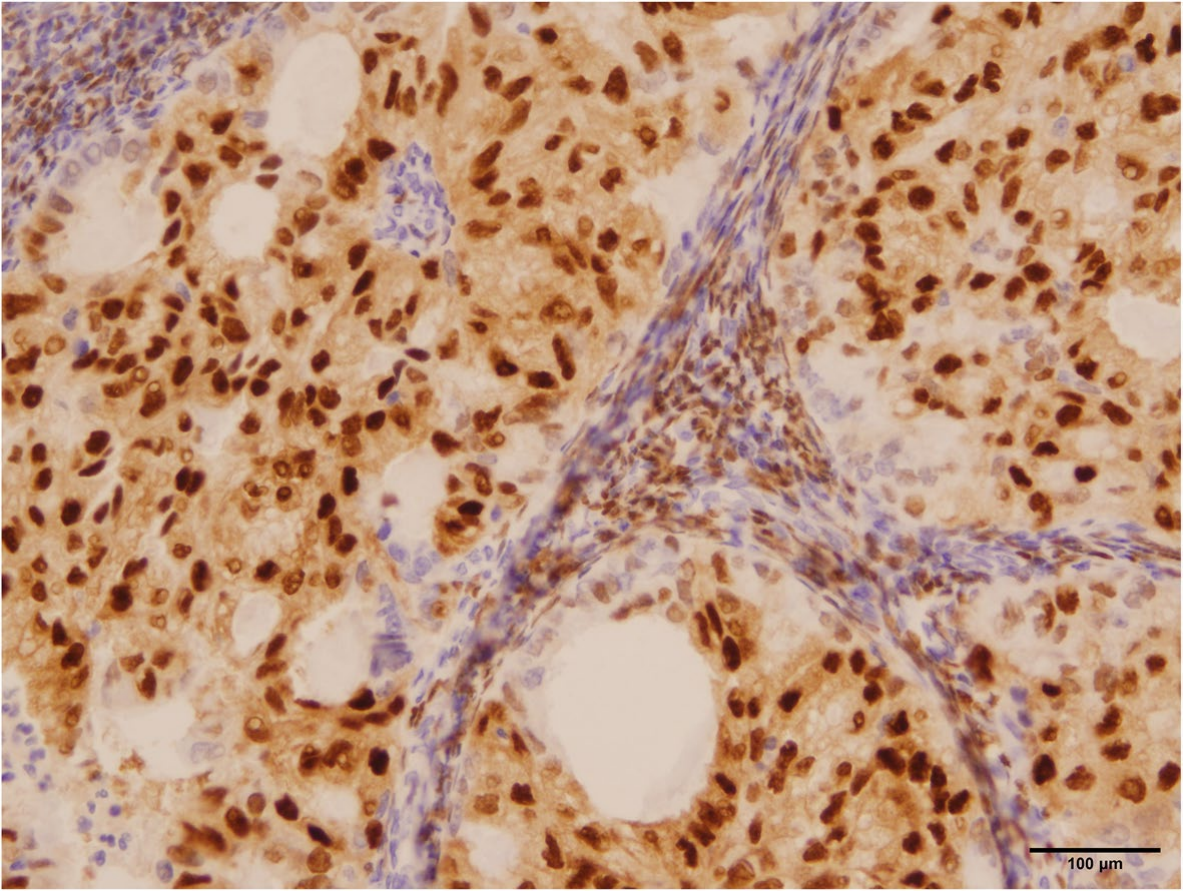
Endometrioid adenocarcinoma with PR strong positive (200 ×)

**Fig. 5:**
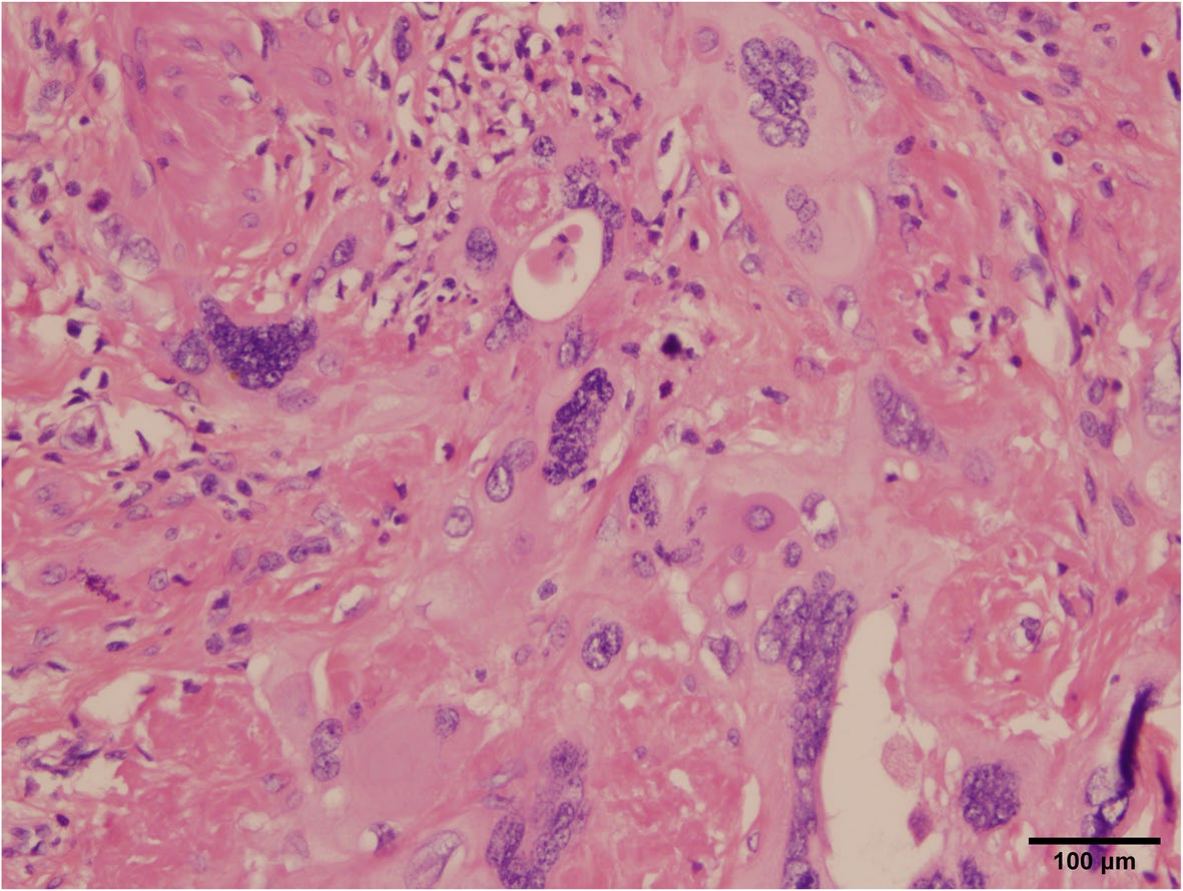
Residual trophoblast cells are seen between the myometrial walls of the uterus (200 ×)

**Fig. 6:**
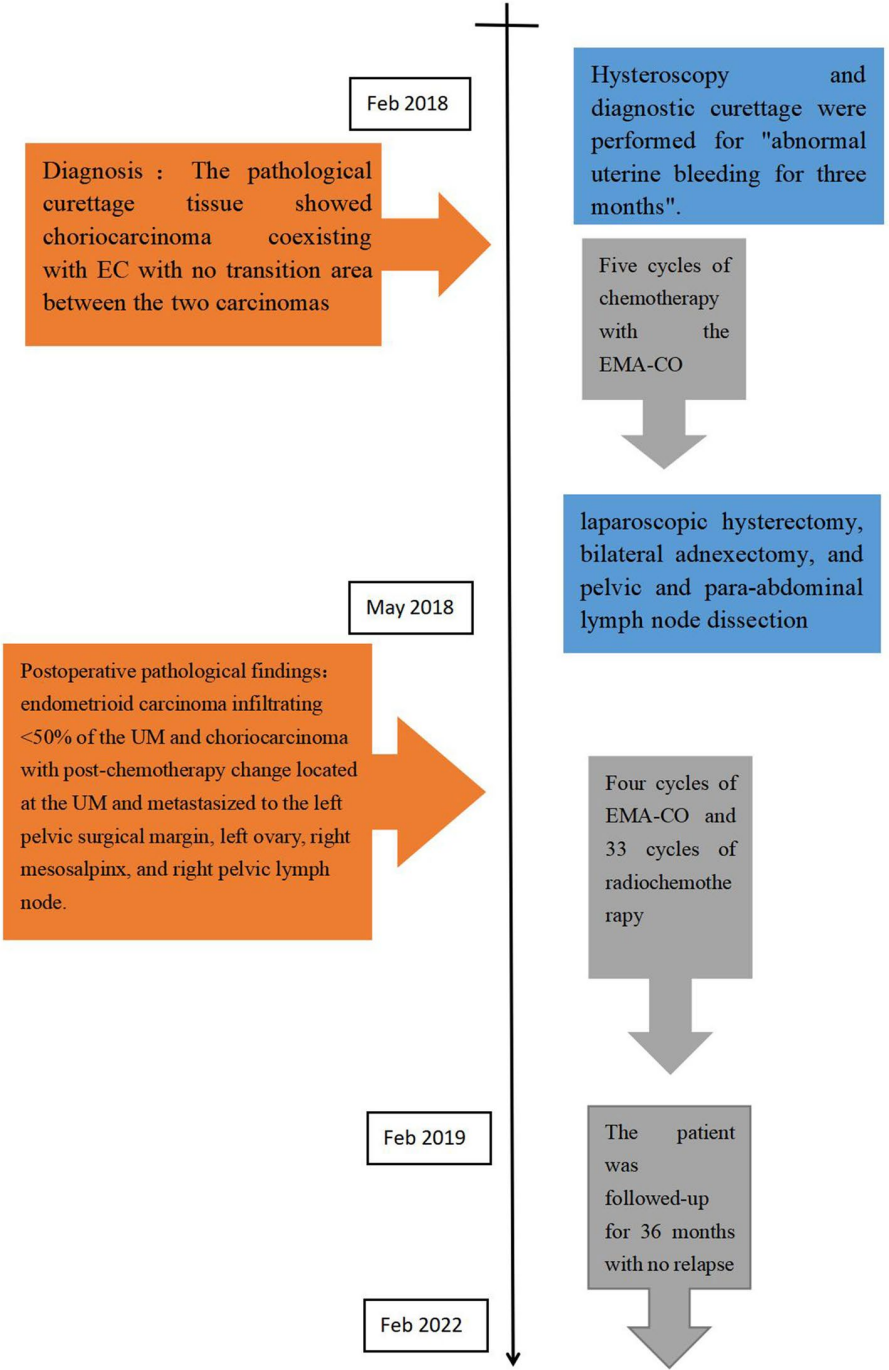
Timeline

A 38-year-old Han Chinese woman underwent a hysteroscopy 49 months ago with abnormal uterine bleeding for the past 3 months. She is an unmarried, sexless, childless, ordinary employee. She usually had dysmenorrhea. There was abnormal uterine bleeding and no other discomfort in the three months prior to the consultation. Uterus enlarged as if more than 3 months pregnant. Previous hysteroscopy revealed a 6 × 4 cm mass protruding from the anterior uterine wall. The mass was resected, and uterine cavity curettage was performed. The patient then presented after which the patient was transferred to our hospital, and a histopathological examination showed that the mass was a choriocarcinoma (Fig. 1). EC immunohistology showed estrogen receptor (+ + +), progesterone receptor (+ + +), and P16( +). The pathological curettage tissue showed choriocarcinoma coexisting with EC with no transition area between the two carcinomas (Fig. 2). Immunohistology showed inhibin A( +) and human chorionic gonadotropin [hCG]( +). Ultrasonography showed an enlarged uterus with a slightly more echoic mass of 5.3 × 5 cm in the uterine myometrium (UM), sufficient blood flow and clear boundary, and the uterine endometrium with uneven thickness and echo. Pulmonary computed tomography (CT) revealed five scattered 0.2 − 0.4 nodules, suspected to be metastatic carcinoma. Cranial, pelvic, and abdominal CTs showed no abnormal findings. The serum β-hCG level was 36,774.5mIU/mL and EMA-CO (etoposide, methotrexate, actinomycin D, cyclophosphamide, and vincristine) was administered to the patient. After five cycles of therapy, the β-hCG level decreased to 35.3 mIU/mL. Pulmonary CT revealed that the nodules had disappeared. Furthermore, the ultrasound showed a 5.0 × 3.8 × 4.9 cm mass in the UM with reduced blood flow. She developed liver function abnormalities after her third chemotherapy treatment. AST(Aspartate aminotransferase) was elevated to 199 U/L. After treatment, AST was reduced to 62 U/L. We performed a laparoscopic hysterectomy, bilateral adnexectomy, and pelvic and para-abdominal lymph node dissection. Pathological examination after surgery showed no obvious abnormal uterine lesions (Fig. 3). Microscopically, there were two different types of carcinomas: endometrioid carcinoma infiltrating < 50% of the UM (Fig. 4), and choriocarcinoma (Fig. 5) with post-chemotherapy change located at the UM and metastasized to the left pelvic surgical margin, left ovary, right mesosalpinx, and right pelvic lymph node. Postoperatively, the serum β-hCG level decreased to the normal range. Four cycles of EMA-CO and 33 cycles of radiochemotherapy (The total radiation dose was 51.7 Gy) were administered after laparoscopic surgery. The patient was followed-up for 40 months with no relapse. The timeline is as follows (Fig. 6).

## Discussion

Trophoblastic differentiation rarely exists in other organs, such as stomach and breast, and in gynecological cancer [3–5]. Since Civantos first reported uterine carcinoma with trophoblastic differentiation in 1972, only 24 cases have been reported to date [6]. In 1987, Savage et al. first reported ECs with trophoblastic differentiation [7]. Since then, ten cases of EC with trophoblastic differentiation have been reported, which is the most common non-trophoblastic component [6]. Other types such as adenocarcinoma, dedifferentiated EC, clear cell carcinoma, endometrial serous carcinoma, and carcinosarcoma have also been reported. Gao et al. reported the first case of choriocarcinoma coexisting with EC. Our case involved two independent carcinomas with no transitional zone between the two, and, therefore, considered ascoexisting.

EC with trophoblastic differentiation is a rare disease characterized by rapid tumor growth, early metastasis, and resistance to chemotherapy. It is important for clinicians and pathologists to identify trophoblastic differentiation in ECs because of poor outcomes. As in our report, abnormal uterine bleeding is the most common clinical symptom that is not specific to this disease. Immunochemical studies are usually required to verify the diagnosis. The β-hCG level was used as a marker for diagnosis and monitoring the treatment effect and relapse in trophoblastic gestational disease and germ cell tumors. Out of 24 cases of uterine carcinoma with trophoblastic differentiation, 23 were reported to have preoperative and postoperative β-hCG elevation, and β-hCG level fluctuated with recurrence [6]. The elevated β-hCG level in our case helped us consider the existence of trophoblastic differentiation, and was used to monitor the treatment effect in our patients.

The hypothesis for trophoblastic differentiation in ECs is as follows: germ cells that fail to complete their migration to the gonads undergo malignant transformation, germ cells outside the urogenital ridge that fail to undergo apoptosis transform into choriocarcinoma, and epithelial cells retrodifferentiate or dedifferentiate into choriocarcinoma [8].

Horn et al. reported two types of uterine carcinoma with trophoblastic differentiation: one was related to gestational choriocarcinoma with a high level of β-hCG and distant metastatic lesions with poor prognosis, and the other represented the characteristics of ECs containing only a few syncytiotrophoblastic giant cells secreting low levels of β-hCG with no distant metastatic lesions and a better prognosis [9]. The prognosis of the disease is related to the components of EC and choriocarcinoma. Therefore, the treatment should treat both components. Our patient responded well to the EMA-CO, and we administered surgery and postoperative radiochemotherapy. She was disease-free 40 months after the treatment ended.

## Conclusion

Here, we report a rare uterine choriocarcinoma coexisting with EC and reviewed the clinicopathological characteristics of endometrial carcinoma with trophoblastic differentiation reported previously in literature. Our case adds important insights to this rare disease and may help inspire additional studies in the future.

## Data Availability

The data supporting the conclusions of this article is available from corresponding author.

## Abbreviation

EC: Endometrial carcinoma
hCG: Human chorionic gonadotropin
UM: Uterine myometrium
CT: Computed tomography
EMA-CO: Etoposide, methotrexate, actinomycin D, cyclophosphamide, and vincristine
AST: Aspartate aminotransferase
